# Detection of circulating natural antibodies against CD25, MUC1, and VEGFR1 for early diagnosis of non‐small cell lung cancer

**DOI:** 10.1002/2211-5463.12878

**Published:** 2020-05-25

**Authors:** Siqi Liu, Xuan Zhang, Quanhang Jiang, Tingting Liang

**Affiliations:** ^1^ Second Hospital of Jilin University Changchun China; ^2^ First Hospital of Jilin University Changchun China

**Keywords:** CD25, MUC1, natural antibody, non‐small cell lung cancer, VEGFR1

## Abstract

We previously demonstrated that a deficiency of natural antibodies against CD25, Mucin 1 (MUC1), and vascular endothelial growth factor receptor 1 (VEGFR1) could contribute to high risk of non‐small cell lung cancer (NSCLC). This study was designed to investigate whether natural IgG antibodies against POU domain class 5 transcription factor 1 (POU5F1), tumor necrosis factor‐α (TNF‐α), and the combination of CD25, VEGFR1, and MUC1 could play an anti‐tumorigenic role against developing NSCLC. An ELISA was developed in‐house to examine plasma IgG against peptide antigens derived from POU5F1, TNF‐α, and a combination of peptide antigens derived from CD25, MUC1, and VEGFR1 in 211 patients with NSCLC and 200 healthy controls. Mann–Whitney *U* test demonstrated that plasma IgG levels for the combination of peptide antigens derived from CD25, MUC1, and VEGFR1 were significantly lower in NSCLC patients than control subjects (*Z* = −12.978, *P < *0.001) although plasma levels of IgG antibodies for POU5F1 and TNFα were not significantly changed. The in‐house ELISA made with the CD25‐MUC1‐VEGFR1 combination had a sensitivity of 49.6% against a specificity of 95% to detect early‐stage NSCLC. In conclusion, natural antibodies against the combination of CD25, VEGFR1, and MUC1 may be an effective biomarker for early diagnosis of NSCLC.

## Graphical abstract

Group I as an early stage (stages 1 and 2A, *n* = 121), group II as stage 2B (*n* = 41), and group III as stages 3 and 4 (*n* = 49). ROC curve analysis showed that the CD25‐MUC1‐VEGFR1 combination‐based ELISA showed the largest AUC of 0.883 (95% CI 0.844–0.923) with a sensitivity of 49.6% against the specificity of 95.0% in patients with early‐stage NSCLC. Natural antibodies against the combination of CD25, VEGFR1, and MUC1 may be an effective biomarker for early diagnosis of NSCLC.

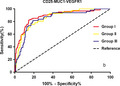

**Abbreviations**
ADCC: antibody‐dependent cellular cytotoxicity
CSLC: cancer stem‐like cell
CV: coefficients of variation
ELISA: enzyme‐linked immunosorbent assay
MUC1: mucin 1
NC: negative control
NSCLC: non‐small cell lung cancer
OD: optical density
PC: positive control
POU5F1: POU domain class 5 transcription factor 1
QC: quality control
SBR: specific binding ratio
SD: standard deviation
TAA: tumor‐associated antigen
TNF‐α: tumor necrosis factor‐α
Treg: regulatory T cells
VEGF: vascular endothelial growth factor
VEGFR1: VEGF receptors 1

According to the 2018 global cancer statistics report, lung cancer is the tumor with the highest incidence and mortality among men, while the third highest morbidity and the second leading cause of cancer death in women worldwide [1]. In 2011, the mortality rate of lung cancer patients in China was as high as 25% [2]. According to histopathological classification, lung cancer has been divided into small cell lung cancer (SCLC) and non‐small cell lung cancer (NSCLC), in which NSCLC accounts for 85% with three subtypes, including adenocarcinoma, squamous cell carcinoma, and large‐cell carcinoma [3]. Although the diagnostic technology and treatment regimens of lung cancer have continuously been improving, the 5‐year survival rate of all combined tumor stages is still less than 20% [4]. Therapeutic efficacy and prognosis of lung cancer are related to pathological stage. Patients with early‐stage lung cancer can have a 5‐year survival rate of 50% [5].

Natural antibodies are a subset of immunoglobulins that are produced by innate B‐lymphocytes without external antigenic stimulation. Natural antibodies can serve as the first line of defense against invading pathogens, removing apoptotic cells and cell debris, and minimizing the inflammatory response. IgM is the main subtype of natural antibodies, but natural IgG and IgA also exist with a range of physiological functions [6, 7]. There is evidence that natural antibodies could destroy transformed cells in the body and contribute to immune surveillance against the development of malignancy [8].

Mucins 1 (MUC1) mainly provide lubrication to the epithelial surfaces and protect the mucosal barriers resisting external physical invasion or biological invasion as a family of glycoproteins widely distributed in various normal mucosal surfaces of the body [9]. MUC1 overexpression and aberrant glycosylation could promote the oncogenesis and metastasis of various malignant tumors including NSCLC [10]. Vascular endothelial growth factor (VEGF) receptor 1 (VEGFR1) promoting angiogenic signaling pathways has been identified as important therapeutic targets in many tumors, including NSCLC [11]. Circulating natural autoantibodies derived from VEGFR1 peptide antigens have been proved to inhibit the proliferation of malignant cell lines derived from hepatocellular carcinoma and pancreatic cancer [8, 12].

POU domain class 5 transcription factor 1 (POU5F1) plays a key role in embryonic development, stem cell pluripotency, proliferative potential, and self‐renewal as the basis of maintaining pluripotent cells. Tumor necrosis factor‐α (TNF‐α) is often found in human tumor tissues and associated with cancer therapy or host response against tumor [13, 14]. However, there are few studies on the relationship between natural autoantibodies against POU5F1 or TNF‐α in NSCLC.

The CD25 is one of the phenotypic markers of CD4^+^ regulatory T (Treg) cells that play an indispensable role in maintaining immune tolerance and regulating immune response [15]. Circulating natural autoantibodies against CD25‐derived peptide antigens have been proved to be altered in several types of cancer including breast cancer and esophageal cancer [16, 17]. Our previous work demonstrated that natural anti‐CD25 antibody might play a significant role in maintaining the homeostasis of the immune system as the detection of circulating natural antibody against CD25 might affect the immune surveillance function and promote tumorigenesis [18].

In a recent study, we have confirmed that the reduction of circulating natural antibodies against MUC1 and VEGFR1 was associated with the carcinogenesis of NSCLC (unpublished data). In this study, accordingly, the combination of three peptide antigens derived from CD25, MUC1, and VEGFR1 was used to develop an enzyme‐linked immunosorbent assay (ELISA) in‐house to obtain additive signals for detection of natural IgG antibodies against these 3 target molecules in NSCLC; we also used the in‐house ELISA to detect natural IgG antibodies for cancer‐related molecules POU5F1 and TNF‐α in this malignancy.

## Materials and methods

### Subjects

A total of 211 patients were recruited with the diagnosis of NSCLC based on radiographic examination and histological confirmation at the Department of Thoracic Surgery, China‐Japan Union Hospital of Jilin University in the period between November 2012 and August 2016. All patients were diagnosed with NSCLC for the first time and did not receive anti‐tumor therapy such as surgery, radiotherapy, chemotherapy, and biotherapy before collection of their plasma samples. Patients with other tumors or autoimmune diseases and those with unqualified blood samples were excluded. These 211 patients included 131 males and 80 females with the mean age of 58.7 ± 8.7 years ranging from 18 to 75 years old. The types of NSCLC were restricted to adenocarcinoma and squamous cell carcinoma only in this study. These NSCLC patients were divided into three subgroups based on the TNM (tumor, node, and metastasis) staging system: group I as an early stage (stages 1 and 2A, *n* = 121), group II as stage 2B (*n* = 41), and group III as stages 3 and 4 (*n* = 49). Blood samples were taken prior to any anticancer treatment. In the same period, 200 healthy volunteers with gender (103 males and 97 females), aged 58.6 ± 9.3 years, and smoking history matched with NSCLC patients were collected in the community. The control subjects did not include those who had either any type of malignant tumors or autoimmune diseases such as autoimmune thyroid disease, pernicious anemia, type 1 diabetes, celiac disease, multiple sclerosis, systemic lupus erythematosus, and inflammatory bowel diseases.

This study was approved by the Ethics Committee of the Second Hospital of Jilin University and in line with the Declaration of Helsinki. All participants were of Chinese Han origin, and all signed written informed consent to participate in the study.

### Detection of plasma IgG levels

An in‐house ELISA was developed to detect plasma natural IgG antibodies against linear peptide antigens derived from POU5F1, TNF‐α, and the combination of CD25, MUC1, and VEGFR1 (the mixture of all 3 peptides in equal concentrations). These peptide antigens were designed by a computational epitope prediction software (http://www.iedb.org) and then synthesized by solid‐phase chemistry with a purity of >95%. Detailed sequence information is given in Table 1. Each peptide antigen was dissolved in 67% acetic acid to obtain a stock solution of 5 mg·mL^−1^ that was diluted in coating buffer (0.1 m phosphate buffer containing 0.15 m NaCl and 10 mm EDTA, pH 7.2) to a working solution of 10 μg·mL^−1^ just before use. Maleimide‐activated 96‐well microplates (Thermo Fisher Scientific, Shanghai, China) were coated with 100 µL of antigen working solution according to manufacturer’s instruction. Plasma IgG antibodies were tested with the antigen‐coated 96‐well microplate, the sample well containing 50 µL plasma diluted 1: 100 in assay buffer in the sample wells, the negative control (NC) well containing 50 µL assay buffer, and the positive control (PC) well containing 50 µL purified IgG solution (50 µg·mL^−1^), which was purified from pooled plasma donated by >1000 healthy subjects [19, 20, 21]. All samples were tested in duplicate, and the variation between the two tests was evaluated by dispersion that is calculated as follows:

$$\text{Dispersion}\;=\left({\text{OD}}_{1}-{\text{OD}}_{2}\right)/\left({\text{OD}}_{1}{\;+\;\text{OD}}_{2}\right).$$

**Table 1:** The sequence information for peptide antigens used for the development of ELISA for antibody test.

Antigen	Sequence (N → C)	NCBI accession	Position (aa)
MUC1	H‐crynltisdvsvsdvpfpfsaqsgah‐OH	NP_002447	149–173
VEGFR1	H‐dlklsctvnkflyrdvtwillrtvnnrtmhysi‐OH	NP_001153502	572–600
CD25	H‐iyhfvvgqmvyyqcvqgyralhrgpaesve‐OH	NP_000408	116–144
POU5F1	keleqfakllkqkritlgytqadvgltc	NP_001272916.1	49–75
TNF‐α	cqlqwlnrranallangvelrdnqlv	NP_000585.2	101–125

If the dispersion was less than 10%, the result was valid; while the dispersion was ≥ 10%, the result was invalid and the test should be repeated. The specific binding ratio (SBR) was used to represent a relative level of plasma IgG, and the SBR was calculated using the optical density (OD) as follows:

$$\text{SBR}\;=\left({\text{OD}}_{\text{sample}}-{\text{OD}}_{\text{NC}}\right)/\left({\text{OD}}_{\text{PC}}-{\text{OD}}_{\text{NC}}\right).$$

To assess the reproducibility of the in‐house ELISA, pooled plasma collected from >30 unrelated healthy donors, called quality control (QC) sample, was tested on every plate. The inter‐assay deviation was estimated with SBR from QC sample test. The coefficient of variation (CV) was used to represent the reproducibility (Table 2).

**Table 2:** The reproducibility of the in‐house ELISA in all IgG tests.

Antigen	Mean ± SD (*n*)^a^	CV (%)
POU5F1	2.020 ± 0.206 (20)	10.21
TNF‐α	1.365 ± 0.173 (20)	12.60
CD25‐MUC1‐VEGFR1	0.473 ± 0.043 (20)	9.03

^a^ (*n*) represents the number of plates tested.

### Statistical analysis

All antibody test data were expressed as means ± standard deviation (SD). Mann–Whitney *U* test was used to examine the differences in plasma IgG levels between patients with NSCLC and healthy controls due to the skewed distribution of plasma antigen‐specific IgG levels based on Kolmogorov–Smirnov one‐sample test (Table 3). Receiver operating characteristic (ROC) curve analysis was performed to work out the area under the ROC curve (AUC) with 95% confidence interval (CI) and the sensitivity of anti‐CD25 IgG assay against a specificity of >95%.

**Table 3:** Kolmogorov–Smirnov test for a normal distribution of plasma IgG level.

IgG	Skewness	Kurtosis	*P* *, a
POU5F1			
Patient	3.307	14.705	＜0.001
Control	3.625	18.398	＜0.001
TNF‐α			
Patient	0.968	2.121	<0.001
Control	0.180	2.551	<0.001
CD25‐MUC1‐VEGFR1			
Patient	0.754	0.690	0.903
Control	−0.083	0.013	0.001

^*^ *P *＞ 0.05 was considered to be normally distributed.

## Results

The demographic information and clinical characteristics of all subjects recruited have been described in our previous publication [19].

The in‐house ELISA made with the CD25‐MUC1‐VEGFR1 combination showed significantly lower IgG levels in NSCLC patients than control subjects (*Z *= −12.978, *P* < 0.001), and both male and female patients contributed to the decrease in plasma IgG levels, while plasma anti‐POU5F1 IgG levels were significantly lower in female patients with NSCLC than female control subjects (*Z *= −2.477, *P* = 0.013) (Table 4). In addition, decreased IgG against POU5F1 and the CD25‐MUC1‐VEGFR1 combination levels mainly occurred in patients with early‐stage NSCLC (Table 5). There was a significant decrease in plasma IgG levels for CD25‐MUC1‐VEGFR1 combinations in patients with either adenocarcinoma or squamous cell carcinoma while a significant decrease in plasma IgG levels for POU5F1 in patients with adenocarcinoma (Table 6).

**Table 4:** The levels of plasma IgG against peptide antigens derived from specific target molecules in patients with NSCLC and control subjects.

IgG	Gender	Patients	Controls	*Z* ^*^, ^a^	*P* ^b^
Pou5F1	Male	0.760 ± 0.556 (131)	0.802 ± 0.623 (103)	−0.803	0.422
	Female	0.692 ± 0.581 (80)	0.811 ± 0.562 (97)	−2.477	0.013
	Both	0.734 ± 0.565 (211)	0.806 ± 0.593 (200)	−2.249	0.025
TNF‐α	Male	1.084 ± 0.325 (131)	1.037 ± 0.318 (103)	−0.366	0.715
	Female	1.066 ± 0.371 (80)	1.123 ± 0.331 (97)	−1.270	0.024
	Both	1.077 ± 0.342 (211)	1.079 ± 0.327 (200)	−0.713	0.476
CD25‐MUC1‐VEGFR1	Male	0.370 ± 0.101 (131)	0.520 ± 0.121 (103)	−8.635	<0.001
	Female	0.346 ± 0.100 (80)	0.555 ± 0.113 (97)	−9.453	<0.001
	Both	0.361 ± 0.101 (211)	0.537 ± 0.118 (200)	−12.978	<0.001

Plasma IgG levels are expressed as mean ± SD in SBI, with *n* = 211 in the patient group (131 males and 80 females) and *n* = 200 in the control group (103 males and 97 females).

^a^ Mann–Whitney *U* test (two‐tailed).

^b^ *P* < 0.017 was considered statistically significant based on the Bonferroni correction.

**Table 5:** The levels of plasma IgG against peptide antigens derived from specific target molecules in subgroups of NSCLC.

IgG	Group^a^	Patient（*n*）	Control（*n*）	*Z* ^b^	*P* ^c^
POU5F1	I	0.735 ± 0.640 (121)	0.806 ± 0.593 (200)	−2.463	0.014
	II	0.678 ± 0.369 (41)	0.806 ± 0.593 (200)	−1.340	0.180
	III	0.778 ± 0.503 (49)	0.806 ± 0.593 (200)	−0.392	0.695
TNF‐α	I	1.031 ± 0.338 (121)	1.079 ± 0.327 (200)	−1.928	0.054
	II	1.137 ± 0.354 (41)	1.079 ± 0.327 (200)	−0.738	0.461
	III	1.142 ± 0.330 (49)	1.079 ± 0.327 (200)	−0.876	0.381
CD25‐MUC1‐VEGFR1	I	0.347 ± 0.107 (121)	0.537 ± 0.118 (200)	−11.504	＜0.001
	II	0.376 ± 0.087 (41)	0.537 ± 0.118 (200)	−7.279	＜0.001
	III	0.384 ± 0.093 (49)	0.537 ± 0.118 (200)	−7.507	＜0.001

Plasma IgG levels were expressed as mean ± SD in SBI.

^a^ Group I for stages 1and 2A, group II for stage 2B, and group III for stages 3 and 4.

^b^ Mann–Whitney *U* test (two‐tailed).

^c^ *P* < 0.017 was considered statistically significant based on the Bonferroni correction.

**Table 6:** The levels of plasma IgG against peptide antigens derived from specific target molecules in two histological types of NSCLC. AC, adenocarcinoma; SCC, squamous cell cancer.

IgG	Type	Patient (*n*)	Control (*n*)	*Z* ^a^	*P* ^b^
POU5F1	SCC	0.775 ± 0.637 (87)	0.806 ± 0.593 (200)	−1.015	0.310
	AC	0.705 ± 0.509 (124)	0.806 ± 0.593 (200)	−2.503	0.012
TNF‐α	SCC	1.105 ± 0.347 (87)	1.079 ± 0.327 (200)	−0.058	0.954
	AC	1.058 ± 0.339 (124)	1.079 ± 0.327 (200)	−1.093	0.275
CD25‐MUC1‐VEGFR1	SCC	0.375 ± 0.106 (87)	0.537 ± 0.118 (200)	−9.413	<0.001
	AC	0.351 ± 0.097 (124)	0.537 ± 0.118 (200)	−11.639	<0.001

Plasma IgG levels are expressed as mean ± SD in SBI.

^a^ Mann–Whitney *U* test (two‐tailed).

^b^ *P* < 0.017 was considered statistically significant based on the Bonferroni correction.

**Fig. 1:**
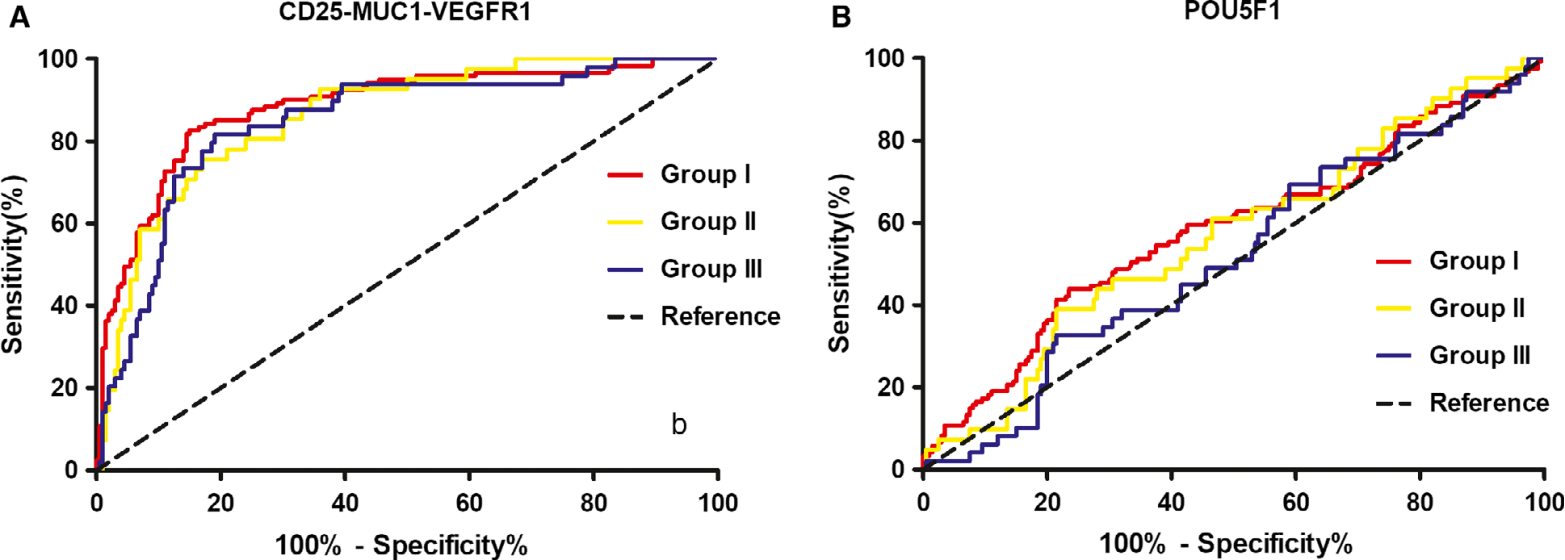
Receiver operating characteristic curve analysis of circulating IgG levels in three subgroups of NSCLC. Group I for stages 1 and 2A, group II for stage 2B, and group III for stages 3 and 4. (A) The CD25‐MUC1‐VEGFR1 IgG assay with a specificity of 95% showed an AUC of 0.883 (95% CI 0.844–0.923) with a sensitivity of 49.6% in group I, an AUC of 0.861 (95% CI 0.804–0.918) with a sensitivity of 39.0% in group II, and an AUC of 0.846 (95% CI 0.785–0.907) with a sensitivity of 26.5% in group III. (B) The POU5F1 IgG assay with a specificity of 95% showed an AUC of 0.582 (95% CI 0.516–0.648) with a sensitivity of 10.7% in group I, an AUC of 0.566 (95% CI 0.470–0.663) with a sensitivity of 7.3% in group II, and an AUC of 0.518 (95% CI 0.429–0.607) with a sensitivity of 2.0% in group III.

Receiver operating characteristic curve analysis showed that the CD25‐MUC1‐VEGFR1 combination‐based ELISA showed the largest AUC of 0.883 (95% CI 0.844–0.923) with a sensitivity of 49.6% against the specificity of 95.0% in patients with early‐stage NSCLC (Fig. 1).

## Discussion

The present study demonstrated that patients with NSCLC, including both adenocarcinoma and squamous cell carcinoma, had a significant decrease in plasma IgG antibodies against the combination of three peptide antigens derived from CD25, MUC1, and VEGFR1, respectively. Patients with adenocarcinoma mainly contributed to the alteration of plasma anti‐POU5F1 IgG levels. Altered anti‐POU5F1 IgG antibody is likely to be involved in a female population, suggesting that deficiency of anti‐POU5F1 IgG is likely to be indicative of a subgroup of NSCLC. It is worth noting that circulating IgG for the CD25‐MUC1‐VEGFR1 combination had the highest sensitivity of 49.6% against a specificity of 95%, with an AUC of 0.883 (95% CI 0.844–0.923) in early‐stage NSCLC, suggesting that natural antibodies for this combination may be an effective biomarker for early diagnosis of NSCLC.

A decrease in natural antibody levels in the body may be involved in many health conditions, such as cancer, cardiovascular disease, and diabetes [22, 23]. In our research, we observed that deficiency of natural IgG antibodies against the CD25‐MUC1‐VEGFR1 peptide combination was strongly associated with lung cancer and that patients with early‐stage NSCLC were more likely to have low antigen‐specific IgG levels in the circulation (Table 5). The above findings raise the possibility that deficiency of natural anticancer antibodies could contribute to a high risk of developing malignancy as natural antibodies could destroy transformed cells and contribute to immune surveillance against carcinogenesis [8, 24, 25]. Therefore, detection of decreased natural antibody levels in the circulation may be a powerful tool for early diagnosis of malignant tumors including NSCLC.

CD25 is the interleukin‐2 receptor alpha chain (IL2RA) and is highly expressed in Treg cells. Increased number of Treg cells in the circulation and high infiltration in tumor microenvironment have been reported in several types of solid tumors [26]. Decreased anti‐CD25 IgG levels may enhance the activity of Treg cells and immune tolerance, and weaken immune surveillance, leading to immune escape of tumor cells [27, 28]. In a recent study, we found that circulating anti‐CD25 IgG antibody levels were significantly decreased in patients with NSCLC [18]. In combination of tumorigenic mechanism and the expression of MUC1 and VEGFR1 in lung cancer, the combined detection of three individual natural autoantibodies is more valuable in early diagnosis of NSCLC.

A series of studies have shown that a small number of cells in lung cancer have tumor initiation activity, which is called cancer stem‐like cell (CSLC) and may be related to the recurrence of lung cancer after surgery or other anti‐tumor treatments [29, 30]. Abnormal expression of POU5F1 contributes to tumorigenesis and metastasis [31]. Our results suggest that anti‐POU5F1 IgG is likely to be indicative of a subgroup of NSCLC, although the resulting ROC curve has failed to show a diagnostic value.

The previous studies have confirmed that peripheral blood antibodies against tumor‐associated antigens (TAAs) are useful biomarkers for early diagnosis of tumors including lung cancer [32, 33]. Chapman and co‐workers developed an in‐house ELISA made either from 6 TAAs (p53, Nyeso‐1, CAGE, GBU4‐5, Annexin 1, and SOX2) or from 7 TAAs (p53, NY‐ESO‐1, CAGE, GBU4‐5, SOX2, HUD, and MAGE A4) to detect plasma anti‐TAA autoantibodies, but they applied increased levels of plasma anti‐TAA antibodies for early diagnosis of lung cancer [33]. In fact, plasma TAA autoantibody levels could be increased with cancer progression, so that such ELISA tests are unlikely to be sensitive enough for early diagnosis. In function, natural autoantibodies can exert anti‐tumor effects by directly inducing antibody‐dependent cellular cytotoxicity and complement‐dependent cytotoxicity. Because of the effect of the immune amplification, even if the tumor antigen content in peripheral blood is lower than a detectable range, the natural autoantibody can still show a higher concentration, even before tumors are formed. Therefore, natural autoantibodies have more advantages than TAAs in early diagnosis of lung cancer.

This study has a couple of limitations. First, sample size used in this study is rather small, so the sample power is not enough to analyze the results from stage 1A patients. Further investigation should focus on stage 1A of NSCLC with large sample size that will lead to a firm conclusion. Second, a prospective study should be carried out to confirm if people with a low level of circulating IgG for the CD25‐MUC1‐VEGFR1 combination are more likely to develop NSCLC than those with a normal level of such autoantibodies.

## Conclusions

The in‐house ELISA made by the combination of three peptide antigens derived from CD25, MUC1, and VEGFR1 could enhance the sensitivity for detection of natural anticancer antibodies that have a diagnostic value in screening of early‐stage NSCLC.

## Conflict of interest

The authors declare no conflict of interest.

## Author contributions

SL carried out laboratory work, data analysis, and drafting the manuscript. XZ conceived this study and corrected the manuscript. QJ was responsible for identification of patients and healthy controls, and collection of samples and clinical information. TL supervised laboratory work and data analysis.
